# Concomitant Mitral Valve Surgery Versus No Intervention in Patients with Moderate Ischemic Mitral Regurgitation Undergoing Coronary Artery Bypass Grafting: A Propensity Score Analysis

**DOI:** 10.14789/jmj.JMJ22-0021-OA

**Published:** 2023-02-22

**Authors:** JIYOUNG LEE, KAN KAJIMOTO, TAIRA YAMAMOTO, ATSUSHI AMANO, MINORU TABATA

**Affiliations:** 1 Department of Cardiovascular Surgery, Juntendo University School of Medcine, Tokyo, Japan; 1 Department of Cardiovascular Surgery, Juntendo University School of Medcine, Tokyo, Japan

**Keywords:** ischemic MR, coronary artery bypass grafting, cardiac surgery

## Abstract

**Objectives:**

Ischemic mitral valve regurgitation (IMR) in patients undergoing coronary artery bypass grafting (CABG) is associated with worse long-term outcomes. This study aimed to assess the impact of mitral valve repair with CABG in patients with moderate IMR.

**Materials:**

This observational study enrolled 3,215 consecutive patients from the Juntendo CABG registry with moderate IMR and multivessel coronary artery disease who underwent CABG between 2002 and 2017.

**Methods:**

The CABG alone and CABG with mitral valve surgery (MVs) groups were compared. The propensity score was calculated for each patient. Long-term all-cause death, cardiac death, and major adverse cardiac and cerebrovascular events (MACCEs) were compared.

**Results:**

Our database had 101 patients who underwent CABG with moderate IMR. Propensity score matching selected 40 pairs for final analysis. MVs was associated with increased risks of postoperative atrial fibrillation, blood transfusion, and longer hospitalization. Long-term outcomes, including all-cause mortality, cardiac mortality, and the incidence of MACCEs were similar.

**Conclusion:**

Surgical treatment of moderate IMR combined with CABG was related to increased risk of several non-fatal short-term complications when compared to CABG alone, with similar long-term outcomes. Further studies are needed to determine the effects of MVs in patients with moderate IMR and severe coronary artery disease.

## Introduction

Ischemic mitral regurgitation (IMR) is a disorder with poor prognosis caused by ischemic heart disease. The mechanisms of IMR involve mitral leaflet tethering due to papillary muscle displacement and increased interpapillary muscle distance, based on impaired left ventricular (LV) systolic function, LV remodeling and dilatation, and mitral valve annulus dilation^1, 2)^. Thus, IMR is recognized as both a valvular disease and an LV disorder.

IMR severity is changed by preload, afterload, and the development of myocardial ischemia. It is known that LV reverse remodeling with improvement of myocardial ischemia leads to reduction of mitral regurgitation (MR)^2)^. Thus, it is difficult to establish reliable treatment strategies for IMR, especially in cases of moderate IMR, where performing coronary artery bypass grafting (CABG) alone or CABG together with mitral valve surgery (MVs) remains controversial^3, 4)^.

This study compared the short-term and long-term outcomes of CABG alone and CABG together with MVs in patients with moderate IMR.

## Materials and Methods

### Study design

This was a retrospective, observational cohort study of prospectively collected data. The protocol for this study was approved by our institutional research ethics committee (H20-0389). We applied Opt-out method to obtain consent on this study through oral information, without a written document. Consecutive patients who underwent CABG between 2002 and 2017 were examined. Patients who had multivessel coronary artery disease and moderate IMR were selected. Patients were further divided into two groups: those who underwent CABG alone and those who underwent CABG with MVs. Before propensity score matching, baseline characteristics were analyzed for the whole sample cohort. The propensity score was calculated for each patient from the results of multivariate logistic regression analysis. Long-term all-cause death, cardiac death, and incidence of major adverse cardiac and cerebrovascular events (MACCEs) were compared between the two groups. Exclusion criteria included any echocardiographic findings of degenerative (chordal or leaflet) mitral valve disease or ruptured papillary muscles. Patients who had a history of previous cardiac surgery were also excluded from the present study.

### Patient data and follow-up

Patient data, including preoperative characteristics, operative data, and postoperative outcomes, were collected from the Juntendo CABG database. Remote outcomes were collected by serial contact (every 3 years) with patients or their families until September 2018. Study coordinators called participants to ask them about adverse events.

### Study outcomes and definitions

The endpoints to compare the efficacy of the two strategies were hospital outcomes, all-cause death, and MACCEs. Postoperative death was defined as death within 30 days of surgery. Postoperative stroke was defined as a new stroke diagnosed on magnetic resonance imaging (MRI) or computed tomography (CT). Postoperative acute kidney injury was defined by a greater than 50% increase in serum creatinine level from baseline. Cardiac death was defined as death by myocardial infarction, congestive heart failure, arrhythmia, or sudden death. The definition of MACCEs included all-cause death, nonfatal myocardial infarction, target vessel revascularization, heart failure requiring hospital admission, and stroke.

Qualifying transthoracic echocardiography was performed before surgery. MR severity was defined based on the criteria recommended by the American Society of Echocardiography^5)^. If leaflet or chordal abnormalities (i.e., primary MR) coexisted, these cases were excluded from the study.

### Operative procedures

All patients underwent surgery with median sternotomy. In isolated CABG cases, off-pump coronary artery bypass (off-pump CABG), which involves performing CABG on the beating heart without cardiopulmonary bypass, was usually performed.

In CABG and MVs cases, cardiopulmonary bypass was established with ascending aorta and right atrial cannulation. Cardiac arrest was obtained with both antegrade and retrograde blood cardioplegia. The strategies for mitral valve intervention were left to the surgeons’ discretion. Mitral valve repair was performed by restrictive mitral annuloplasty (MAP) using an artificial ring to correct annular dilatation. Mitral valve replacement was performed with preservation of the posterior leaflet or subvalvular apparatus. The techniques of mitral valve repair and the choice of prosthetic valve were determined by the surgeons.

### Statistical analysis

Continuous variables are expressed as means ± standard deviation, and categorical data are tabulated as frequencies and percentages. These data were compared using Student’s t-test or Mann-Whitney U test for continuous variables, and the χ^2^ test or Fisher’s exact test for categorical variables. The propensity score was calculated for each patient from the results of multivariate logistic regression analysis based on preoperative covariates as independent variables with CABG alone vs. CABG plus MVs as binary dependent variables. The following preoperative patient characteristics were the exploratory variables of the logistic regression model: sex, age, BSA, diabetes (yes/no), dyslipidemia (yes/no), hypertension (yes/no), estimated GFR (mL/min/1.73m^2^), history of cerebrovascular accident (yes/no), peripheral artery disease (yes/no), history of myocardial infarction (yes/no), LV ejection fraction, preoperative atrial fibrillation(yes/no).

Short-term postoperative complications were compared between the two groups. Long-term all-cause death, cardiac death, and MACCEs were compared, and the Kaplan-Meier method with the log-rank test was used for these survival analyses. A Cox proportional-hazards model was used to assess the association between the survival time of these events and one or more predictor variables, that were determined with reference to previous other studies. Values of p<0.05 were considered significant. All data were analyzed using SPSS version 23.0 for Windows (SPSS, Chicago, IL).

## Results

### Patients’ characteristics and operative data

A total of 3,215 patients underwent CABG between 2002 and 2017. Of these patients, 101 who had multivessel coronary artery disease and moderate IMR were eligible for the study. These 101 patients were divided into two groups, 60 (59.4%) who underwent CABG alone and 41 (40.6%) who underwent CABG with MVs. Propensity score matching selected 40 pairs for final analysis. The p-value of the Hosmer-Lemeshow test for the model was 0.787, and the c-statistic (area under the ROC curve) was 0.707. The mean propensity score of the CABG alone group was 0.50±0.25, and that of the CABG plus MVs group was 0.58±0.22. A higher propensity score indicated a higher probability of undergoing CABG with MVs at baseline.

Comparison of baseline characteristics between the CABG alone and CABG plus MVs groups before and after propensity score matching is shown in Table 1. Even before propensity score matching, there were no significant differences between the two groups in the variables.

**Table 1:** Baseline characteristics of patients undergoing CABG alone vs. CABG + MVs

	Before matching			After matching		
	CABG (N=60)	CABG + MVs (N=41)	p-value	CABG (N=40)	CABG + MVs (N=40)	p-value
Age (y) mean ± SD	68.9 ± 9.4	68.2 ± 8.6	0.720	69.5 ± 9.6	68.2 ± 8.7	0.536
Sex male, n (%)	48 (80.0%)	33 (80.4%)	0.556	34 (85.0%)	32 (80.0%)	0.562
BSA (m^2^), mean ± SD	1.63 ± 0.14	1.65 ± 0.12	0.478	1.63 ± 0.13	1.65 ± 0.12	0.660
Diabetes mellitus, n (%)	37 (61.6%)	21 (51.2%)	0.314	25 (62.5%)	21 (52.5%)	0.372
HbA1c (%), mean ± SD	6.4 ± 1.1	6.1 ± 1.2	0.225	6.4 ± 1.1	6.1 ± 1.2	0.255
Dyslipidemia, n (%)	42 (70.0%)	24 (58.5%)	0.234	28 (70.0%)	24 (60%)	0.348
Serum TG level (mg/dL), mean ± SD	118.0 ± 59.4	111.8 ± 46.2	0.576	115.0 ± 56.9	112.9 ± 46.2	0.857
Serum LDL cholesterol level (mg/dL), mean ± SD	102.8 ± 38.8	100.8 ± 29.7	0.787	93.6 ± 36.4	100.5 ± 30.0	0.363
Hypertension, n (%)	46 (75.4%)	30 (75.0%)	0.689	34 (85.0%)	30 (75.0%)	0.263
Estimated GFR (mL/min/1.73m^2^), mean ± SD	50.3 ± 27.8	48.6 ±39.2	0.808	48.2 ± 24.7	49.6 ± 30.2	0.850
History of cerebrovascular accident, n (%)	10 (16.6%)	5 (12.1%)	0.373	7 (17.5%)	4 (10.0%)	0.336
Peripheral artery disease, n (%)	8 (13.3%)	7 (17.0%)	0.777	6 (15.0%)	7 (17.5%)	0.765
History of myocardial infarction, n (%)	8 (13.3%)	8 (19.5%)	0.403	5 (12.5%)	8 (20.0%)	0.363
BNP (pg/mL), mean ± SD	760.8 ± 1512.6	613.2 ± 781.0	0.574	504.7 ± 568.6	613.2 ± 781.0	0.480
LVEF (%), mean ± SD	41.1 ± 13.2	37.6 ± 16.5	0.251	37.5 ± 11.2	34.8 ± 14.5	0.382
Preoperative AF, n (%)	4 (6.6%)	7 (17.0%)	0.094	3 (7.5%)	7 (17.5%)	0.181
EuroSCORE II (%), mean ± SD	6.8 ± 8.6	7.5 ± 6.9	0.699	5.9 ± 7.7	7.5 ± 6.9	0.365
Japan SCORE (%), mean ± SD	5.6 ± 11.9	7.5 ± 12.2	0.497	5.5 ± 13.1	7.5 ± 12.2	0.516

Abbreviations: AF, atrial fibrillation; BNP, brain natriuretic peptide; BSA, body surface area; HbA1c, hemoglobin A1c; LVEF, LV ejection fraction; TG, triglycerides

Operative parameters are shown in Tables 2 and 3. Overall, 95% of patients (n=38) in the CABG alone group underwent CABG without cardiopulmonary bypass (off-pump CABG). The number of coronary anastomoses was comparable between the two groups (3.7±1.4 vs. 3.6±1.7; p=0.52). In the CABG alone group, the rate of using bilateral internal thoracic arteries was significantly higher (75.0% vs. 47.5%; p=0.012), and saphenous vein grafts were less likely to be used (40.0% vs. 62.5%; p=0.07). The mean operation time was significantly longer in the CABG plus MVs group than in the CABG alone group.

**Table 2:** Operative data of patients undergoing CABG alone vs. CABG + MVs

	CABG (N=40)	CABG + MVs (N=40)	p-value
Preoperative IABP, n (%)	2 (5.0%)	1 (2.5%)	0.556
Number of grafts selected, mean ± SD	2.7 ± 0.7	2.4 ± 0.7	0.142
Left internal thoracic artery, n (%)	39 (97.5%)	35 (87.5%)	0.090
Bilateral internal thoracic artery, n (%)	30 (75.0%)	19 (47.5%)	0.012
Radial artery, n (%)	3 (7.5%)	2 (5.0%)	0.644
Gastroepiploic artery, n (%)	20 (50.0%)	17 (40.2%)	0.501
Saphenous vein graft, n (%)	16 (40.0%)	25 (60.2%)	0.044
Number of distal anastomoses, mean ± SD	3.7 ± 1.4	3.6 ± 1.7	0.725
Off-pump surgery	38 (95%)	0 (0.0%)	
Operation time duration (min), mean ± SD	300.3 ± 74.4	468.2 ± 124.2	< 0.001
Aorta cross clump time (min), mean ± SD	-	105.8 ± 51.2	
Cardiopulmonary bypass (min), mean ± SD	159.0 ± 14.1	188.3 ± 76.0	0.586

Abbreviations: IABP, intra-aortic balloon pump

**Table 3:** Details of mitral valve procedures

Procedure	CABG + MVs (N=40)
Mitral annuloplasty (MAP) alone, n (%)	17 (42.5%)
MAP + Procedure of MV leaflet or subvalvular apparatus, n (%)	19 (47.5%)
Leaflet edge-to-edge repair, plication, chordal cutting, n (%)	7 (17.5%)
Chordal cutting, n (%)	5 (12.5%)
Papillary muscle approximation (PMA), n (%)	10 (25.0%)
LV reconstruction, n (%)	5 (12.5%)
Mitral valve replacement, n (%)	4 (10.0%)

Preoperative echocardiogram data are shown in Table 4. The preoperative echocardiogram at baseline showed no significant differences between the two groups in LV ejection fraction (LVEF) (39.3%±12.8% vs. 36.7%±15.5%), LV size (LVDd, Ds, EDVI, ESVI), mitral annular diameter size, and degree of IMR or tethering (ERO and RV(PISA), tenting height).

**Table 4:** Preoperative echocardiography of patients undergoing CABG alone vs. CABG + MVs

	CABG (N=40)	CABG + MVs (N=40)	p-value
Left atrial diameter (mm), mean ± SD	44.7 ± 6.7	47.3 ± 8.8	0.192
Mitral annulus diameter (mm), mean ± SD	34.1 ± 5.5	31.5 ± 3.8	0.105
LV diastolic dimension (mm), mean ± SD	58.5 ± 7.3	59.2 ± 7.7	0.735
LV systolic dimension (mm), mean ± SD	48.6 ± 10.0	47.6 ± 11.5	0.720
LV EDVI (mL/m2), mean ± SD	101.9 ± 38.0	110.7 ± 41.3	0.388
LV ESVI (mL/m2), mean ± SD	64.6 ± 31.5	74.6 ± 39.8	0.278
ERO (PISA), mean ± SD	0.26 ± 0.06	0.26 ± 0.08	0.827
RV (PISA) (mL), mean ± SD	39.3 ± 10.8	42.5 ± 15.7	0.443
Tenting height (mm), mean ± SD	10.8 ± 2.3	9.8 ± 2.0	0.178
LVEF (%), mean ± SD	37.5 ± 11.2	34.8 ± 14.5	0.382

Abbreviations: Dd, diastolic dimension; Ds, systolic dimension; EDVI, end-diastolic volume index; ERO, effective regurgitant orifice; ESVI, end-systolic volume index; LV, left ventricular; LVEF, LV ejection fraction; RV, regurgitant volume

### Short-term outcomes

The postoperative short-term outcomes are shown in Table 5. There was no early death in the CABG alone group, and only one death in the CABG plus MVs group (1.25% of all matched patients). The incidences of stroke, respiratory failure or pleural effusion, acute kidney injury, and re-exploration for bleeding were similar between the two groups. When compared with the CABG alone group, the incidences of postoperative atrial fibrillation (37.5% vs. 62.5%, p=0.025) and postoperative blood transfusion (40.0% vs. 75.0%, p=0.002) were higher, and the mean lengths of ICU stay and hospital stay (3.2 days vs. 6.0 days, p=0.084, 13.6 days vs. 18.1 days, p=0.035, respectively) were much longer in the CABG plus MVs group.

**Table 5:** Hospital outcomes of patients undergoing CABG alone vs. CABG + MVs

A) All 101 patients			
	CABG (N=60)	CABG + MVs (N=41)	p-value
In-hospital death, n (%)	3 (5.0%)	2 (%)	0.676
Postoperative AF, n (%)	22 (36.6%)	26 (63.4%)	0.007
Postoperative stroke, n (%)	1 (1.6%)	1 (2.4%)	0.650
Respiratory failure / Pleural effusion, n (%)	13 (21.6%)	13 (31.7%)	0.183
Acute kidney injury, n (%)	6 (10.0%)	6 (14.6%)	0.343
Blood transfusion, n (%)	25 (41.6%)	31 (75.6%)	0.001
Re-exploration for bleeding, n (%)	0 (0.0%)	1 (2.4%)	0.406
Length of ICU stay (day), mean ± SD	3.2 ± 3.5	5.8 ± 9.2	0.089
Length of hospital stay (day), mean ± SD	13.4 ± 8.4	17.7 ± 9.8	0.021
B) Matched patients			
	CABG (N=40)	CABG + MVs (N=40)	p-value
In-hospital death, n (%)	0 (0.0%)	1 (2.5%)	0.314
Postoperative AF, n (%)	15 (37.5%)	25 (62.5%)	0.025
Postoperative stroke, n (%)	1 (2.5%)	1 (2.5%)	1.0
Respiratory failure / Pleural effusion, n (%)	6 (15.0%)	12 (30%)	0.108
Acute kidney injury, n (%)	3 (7.5%)	6 (15.0%)	0.288
Blood transfusion, n (%)	16 (40.0%)	30 (75%)	0.002
Re-exploration for bleeding, n (%)	0 (0.0%)	0 (0.0%)	
Length of ICU stay (day), mean ± SD	3.2 ± 4.1	6.0 ± 9.3	0.089
Length of hospital stay (day), mean ± SD	13.6 ± 8.9	18.1 ± 9.6	0.035

Abbreviations: AF, atrial fibrillation; LVEF, left ventricular ejection fraction

### Long-term outcomes

**Figure 1:**
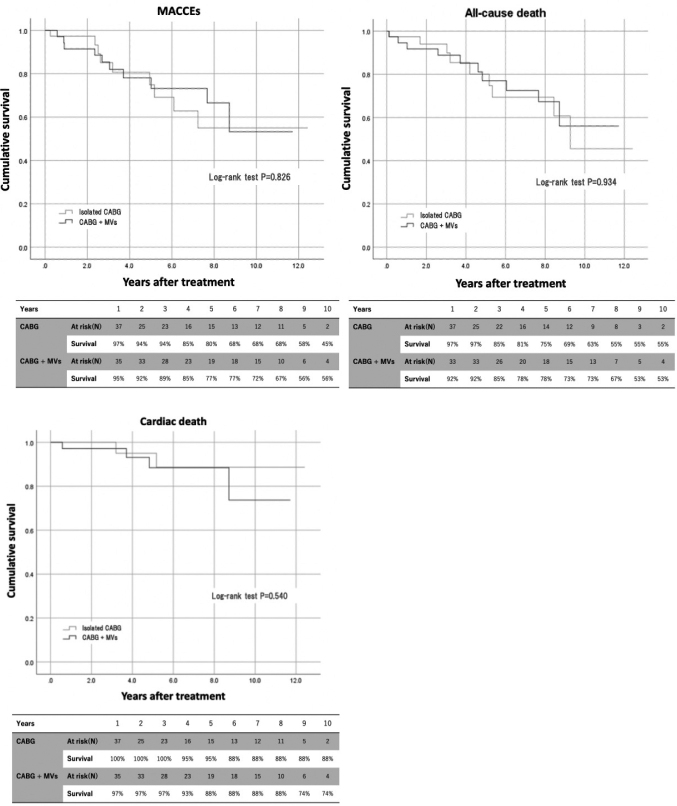
Kaplan-Meier survival curves for all-cause death, cardiac death, MACCEs

The median follow-up period was 3.6 (1.4-8.1) years. Cumulative event-free curves with log-rank tests are shown in Figure 1. Long-term all-cause mortality, cardiac mortality, and the incidence of MACCEs were similar between the groups. There was no reoperation in both groups. Five-year survival rates for patients in the CABG alone group and in the CABG plus MVs group were 80% and 77%, respectively. Concomitant MVs was not associated with an increased risk of long-term all-cause death or MACCE. Table 6 shows Hazard ratios of several risk factors for long-term all-cause mortality, cardiac mortality, and the incidence of MACCEs, calculated by a Cox proportional-hazards model. History of cerebrovascular accident was an independent risk factor for these long-term events.

**Table 6:** Multivariable predictors for all-cause mortality, cardiac death, and MACCE

	Hazard ratios	95% confidence intervals	p-values
All-cause death			
Combined MV surgery	0.96	0.56 - 1.64	0.886
Age	1.02	0.98 - 1.07	0.179
Sex male	1.28	0.53 - 3.07	0.570
Diabetes mellitus	1.20	0.66 - 2.18	0.530
Peripheral vascular disease	0.90	0.37 - 2.17	0.815
History of cerebrovascular accident	2.51	1.07 - 5.88	0.047
Estimated GFR	0.99	0.98 - 1.01	0.828
LVEF	0.72	0.37 - 1.38	0.327
			
Cardiac death			
Combined MV surgery	0.97	0.59 - 1.59	0.917
Age	1.02	0.98 - 1.06	0.176
Sex male	1.30	0.58 - 2.87	0.506
Diabetes mellitus	1.06	0.62 - 1.82	0.819
Peripheral vascular disease	1.15	0.56 - 2.36	0.695
History of cerebrovascular accident	2.67	1.21 - 5.88	0.022
Estimated GFR	1.00	0.99 - 1.01	0.804
LVEF	0.67	0.37 - 1.18	0.164
			
MACCEs			
Combined MV surgery	0.96	0.56 - 1.65	0.897
Age	1.01	0.97 - 1.06	0.510
Sex male	1.16	0.50 - 1.77	0.720
Diabetes mellitus	1.08	0.59 - 1.97	0.799
Peripheral vascular disease	1.18	0.53 - 2.61	0.678
History of cerebrovascular accident	2.63	1.17 - 5.93	0.027
Estimated GFR	1.00	0.99 - 1.01	0.426
LVEF	0.84	0.44 - 1.62	0.618

Abbreviations: MACCEs, major adverse cardiac and cerebrovascular event

## Discussion

This propensity matched study evaluated the short-term and long-term outcomes of CABG alone and CABG plus MVs in patients with moderate IMR. In this study, combined mitral valve treatment was associated with increased risks of several postoperative adverse events (atrial fibrillation, postoperative blood transfusion, and prolonged lengths of ICU stay and hospital stay), but short-term mortality and the incidences of stroke, acute kidney injury, bleeding, and respiratory failure were similar between the two groups.

Some studies have shown that off-pump CABG, compared with on-pump CABG, was associated with lower risks of mortality, stroke, renal failure, RBC transfusion, prolonged ventilation, inotropic support, and intra-aortic balloon pumping support, especially in higher risk patients^6-8)^. In the present study, despite the fact that almost all isolated CABGs were performed without cardiopulmonary bypass, short-term mortality, cerebrovascular events, kidney injury, and respiratory failure were similar between two groups. This result suggests that combined MVs, which needs cardiopulmonary bypass, can be performed in patients with moderate IMR as safely as off-pump CABG. Probable explanations for this finding are improvements of cardiopulmonary bypass, cardioplegia, anesthesia, and perioperative medications^9, 10)^.

Furthermore, during this study’s long-term follow-up period, both groups were similar in all-cause death, cardiac death, and the incidence of MACCEs. In addition, postoperative reverse remodeling was observed in both groups. These findings were consistent with two recent prospective, randomized trials^11, 12)^. These studies, which randomized 301 patients with moderate IMR to undertake isolated CABG or CABG plus MAP, showed that moderate or severe residual MR was more frequently observed in patients undergoing CABG alone, but showed no difference between the two procedures in LV reverse remodeling, mortality, overall adverse events, and readmissions at two years after operation.

On the other hand, these results contradicted other previous studies^13, 14)^. Fattouch and colleagues reported the first prospective, randomized study of moderate IMR, comparing patients with undergoing CABG alone or CABG plus MAP for an average of 32 months^13)^. They showed that the addition of MAP to CABG significantly improved LV reverse remodeling, severity of MR, and NYHA functional class compared with CABG alone. Similarly, the Randomized Ischemic Mitral Evaluation (RIME) trial^14)^, which was another randomized study of moderate IMR, demonstrated that oxygen consumption, severity of MR, plasma B-type natriuretic peptide levels, and LV reverse remodeling were improved in patients assigned to CABG plus MVs compared to those assigned to CABG alone. These studies proved the beneficial effects of CABG plus MVs in patients with moderate IMR by showing that concomitant mitral valve restoration not only reduced the degree of severity of mitral valve regurgitation, but also provided an improvement in the NYHA functional class. Therefore, in deciding on additional MVs, the presence of symptoms such as dyspnea, shortness of breath, and heart failure, might be important, which means MR bears more clinical and prognostic significance.

Why various results were observed in studies that compared CABG alone and CABG plus MVs for patients with moderate IMR? One can only speculate on these discrepancies between the results of these studies. Since the mechanisms of IMR are various and complicated^1, 2)^, patients’ characteristics at baseline might be heterogeneous and imbalanced among these studies. For example, when comparing the three clinical trials mentioned above, in the study by Fattouch and colleagues^13)^ and in the RIME trail^14)^, patients had significantly higher rates of previous MI and larger LV size, and remodeling was more advanced than in the CTSN trial^12)^ at baseline. Postoperative echocardiogram showed that the degree of reverse LV remodeling was greater in the first two studies than in the CTSN trial. In each of the three studies, the combined procedure group had a higher rate of postoperative residual or recurrent MR, but there was no significant difference in mortality. What can be inferred from these studies is that combined MVs for moderate IMR is not associated with increased long-term survival, instead, it may contribute to improvement of other endpoints, including LV reverse remodeling, MR grade, and cardio-humoral factors, especially in patients with larger ventricles and advanced LV remodeling at baseline. Conversely, patients with smaller ventricles at baseline can obtain the benefit of LV reverse remodeling from CABG alone to the same extent as CABG plus MVs.

About other factors to predict LV reverse remodeling after CABG, several studies have demonstrated that the presence of preoperative viable myocardium is closely related to improvements in LV reverse remodeling and downgrading of IMR. Reliable improvement in MR after surgery was limited to those who had viable myocardium and less LV dyssynchrony between papillary muscles for patients with moderate IMR who underwent isolated CABG^15, 16)^. Conversely, patients whose cardiac muscle has suffered ischemic changes for a long period and are less likely to have sufficient viable cardiac muscle, and those who have fewer or no bypass target to the posterior-inferior-lateral area are not better off with isolated CABG. Unfortunately, myocardial viability were not included in many randomized trial mentioned above, and also in this analysis.

Therefore, to decide whether to perform MVs at the time of CABG in patients with moderate IMR, we should know which patient may benefit most from CABG plus MVs rather than CABG alone. Preoperative evaluation of several factors, such as MR severity, left atrium enlargement or mitral annulus dilatation, LV size and remodeling degree, severity of LV dysfunction, presence of LV scar tissue or myocardial viability, and presence of an efficient bypass target to the posterior-inferior- lateral area, might be helpful to determine the surgical plan^3, 12)^. To assess these variables, preoperative stress echocardiography and cardiac catheterization, single photon emission computed tomography (SPECT), and functional cardiac MRI are preferred, if possible^17, 18)^. However the ability to predict MR response to CABG alone is still poor because there has been insufficient validation.

In addition, we should also consider patient factors or comorbidities such as age, frailty, number of bypass targets, renal failure, respiratory failure, peripheral artery disease, thoracic aortic calcification, arteriosclerosis, previous cerebral infarction, etc. The surgeon’s experience with MVs is important as well, because the effect of mitral valve repair depends on the accuracy with which it is performed, and if the surgeon lacks experience with mitral valve repair, not adding MVs may be better in order to decrease the duration and total risk of the operation.

This study had several limitations that could affect the interpretation of our findings. Despite the prospective collection of operative data, this was a retrospective study in a single institution. Although propensity score analysis was performed, the number of patients was small because moderate IMR is an uncommon disease. This study was susceptible to various sources of bias. The decision for MVs was not decided based on specific preoperative criteria, and the surgeon determined the surgical details of CABG or MVs, based on various center/patient-specific factors. In addition, myocardial variables such as local wall motion score or viability were not included in this analysis, therefore the relationship between myocardial variables and long-term prognosis were not investigated in this study. Finally, another limitation is that clinical outcomes related to heart-failure symptoms such as NYHA class were not assessed, and neither were improvements in patients’ quality of life and physical function, which might be a benefit of a lower incidence of postoperative MR by additional MVs, as some studies have reported.

In conclusion, the present study showed that surgical treatment of moderate IMR combined with CABG could be performed as safe as isolated CABG (off-pump CABG). In contrast, surgical treatment of moderate IMR did not improve long-term outcomes, including all-cause mortality, cardiac mortality, and the incidence of MACCEs. In patients with moderate IMR, the indication for concomitant mitral valve surgery and the problem of whether to replace or repair, and whether to add subvalvular and leaflet approaches to ring annuloplasty, remain controversial. Further studies are necessary to determine the effects of MVs and optimal selection of patient with moderate IMR with severe coronary artery disease.

## Funding

The authors have no financial support for the research.

## Author contributions

JL analyzed and interpreted the patient data, and was a major contributor in writing the manuscript. KK contributed to the design of the work, and revised the manuscript critically. YT was a major contributor in the acquisition of data for the work. All authors read and approved the final manuscript.

## Conflicts of interest statement

The authors declare that there are no conflicts of interest.
